# Collectivist culture, environmental regulation and pollution emissions: evidence from China

**DOI:** 10.3389/fpsyg.2023.1300601

**Published:** 2024-01-05

**Authors:** Li Zhang, Miao Zhang, Jie Jia, Xu Peng, Jiaxuan Zhu, Shibing You

**Affiliations:** School of Economics and Management, Wuhan University, Wuhan, China

**Keywords:** collectivist culture, environmental regulation, pollution reduction, environment governance, fixed-effect model

## Abstract

Collectivist culture serves as a significant cultural foundation in China. It could, to some extent, shape public attitudes toward the environment and thus influence the implementation of related policies. To examine this hypothesis, this study constructs the collectivist culture intensity index for 25 Chinese provinces spanning from 2010 to 2020. Through a fixed-effect model, we explore how the collectivist culture intensity affects pollution emissions in China. The empirical results indicate the significance of collectivism in enhancing emission reduction through environmental regulations. This conclusion remains robust even when excluding the impact of endogeneity concerns by adopting the instrumental variable approach. Heterogeneity analysis shows that collectivism is more effective in enhancing market-based environmental regulations rather than those driven by policies. Further mechanism analysis confirms that green innovation is a crucial pathway through which collectivism influences pollution emissions. These findings here will offer guidance to policymakers when formulating environmental policies for contexts with different regional cultures.

## 1 Introduction

With China’s rapid economic development, environmental protection issues have become more prominent. Fast-paced industrialization and urbanization have led to significant energy consumption and waste emissions, severely impacting environmental quality. Pressing challenges include addressing coal and automobile exhaust emissions, industrial wastewater, and solid waste management, exerting immense pressure on both the ecological environment and public health (Alford and Liebman, 2000). According to the Environmental Performance Index Report (Wendling et al., 2020), China ranked 120th out of 180 countries and regions assessed, reflecting the multitude of environmental challenges to be confronted.

Environmental pollution not only undermines the physical and mental well-being of citizens (Schlenker and Walker, 2011; Chen and Chen, 2018; Weinmayr and Forastiere, 2022), but also carries substantial economic costs (Ito and Zhang, 2020). In response, the Chinese government has made efforts to strike a balance between economic growth and environmental protection (Tu and Chen, 2015), and implemented environmental regulations to enhance the management and control of pollutant emissions (Chen et al., 2021; Luo and Qi, 2021; Dong and Chang, 2023).

However, regulations often fall short of effectively addressing environmental issues due to deficiencies in environmental governance and policy implementation. Economic development pursuit by local officials may take precedence over environmental protection due to promotion incentives (Jia, 2017). Collusion between the government and enterprises can lead to increased pollution emissions after inspection periods (Sun et al., 2022). Relying solely on legislative measures without robust enforcement and supervision is insufficient to significantly curb pollution intensity (Bao et al., 2013). Informal institutions, from the perspective of new institutional economics, encompass a broader range of constraints and exhibit contagious continuity compared to formal regulations (Hu et al., 2017). Therefore, informal institutions can enhance the effectiveness of environmental regulations when formal regulations prove ineffective (Tietenberg, 1998; Xu, 2014).

The informal institutions are the unwritten and widely recognized behavioral norms that develop unconsciously through long-term social interactions (North, 1990). They stem from trust and consensus among individuals. Current research on informal institutions related to environmental regulations mainly centers around factors such as religion and lineage (Bi et al., 2015), trust (He et al., 2015; Yang and Niu, 2023), hometown identification (Hu et al., 2017), and individual consciousness (Boiral et al., 2018). Collectivism is a prominent cultural characteristic worldwide and significantly shapes individual and societal behaviors (Lonner et al., 1980). Given this influence, it is reasonable to hypothesize that individuals from various collectivist cultural backgrounds may display distinct attitudes and behaviors toward the environment, potentially impacting the effectiveness of environmental regulations. Thus, this study aims to investigate how collectivist culture influences environmental regulations as an informal institution.

The characteristic of collectivism is evident in individuals forming close group relationships for mutual protection (Lonner et al., 1980). In collectivist cultures, when an individual’s opinions differ from those of the larger group, consideration is given to the impact of individual behavior on others. As a result, individual interests are often subordinated to the collective well-being. In contrast, in individualistic cultures, individuals tend to prioritize personal achievements or immediate benefits (Bhagat, 2002). Therefore, these two cultures, collectivism and individualism, have different influences on people’s propensity to engage in environmental behaviors.

There is scope for improving the assessment of collectivist culture, not only theoretically but also in quantitative analysis. For instance, relying solely on methods like the rice theory (Talhelm et al., 2014) and dummy variables to measure collectivist intensity might overlook relevant information (Xu et al., 2016; Han et al., 2021). Hence, employing more refined quantitative methodologies is essential to validate the influence of collectivist culture on environmental regulations.

In this study, we aim to explore the combined impact of collectivist culture and individual environmental behaviors on regulatory effects, especially those related to environmental regulations. Our study has several distinct characteristics compared to previous research. Firstly, we have improved the methodology for constructing collectivism indicators based on existing studies. By creating a continuous measure of collectivism at the provincial level in China, we are able to better capture the temporal and spatial heterogeneity of collectivism in the country. Secondly, we have focused specifically on the role of regional collectivist culture in moderating formal environmental regulations, examining the robustness of our findings from multiple perspectives. Lastly, our study has validated the pathways and mechanisms through which collectivism influences pollution emissions.

## 2 Literature review and hypothesis development

### 2.1 Collectivist culture and environmental regulation

China has the strongest collectivist culture in the world (Van de Vliert et al., 2013). Existing research attributes the origins of China’s collectivism to Confucian culture (Bond and Hwang, 1986), widespread diseases (Fincher and Thornhill, 2012), agricultural patterns (Talhelm et al., 2014), climate (Van de Vliert, 2011), and urbanization (Freeman, 1997), among many other factors. And studies acknowledge the presence of regional variations in collectivism within China (Gong et al., 2021).

The impact of regional collectivist culture on environmental regulations and pollution emissions encompasses various dimensions. Firstly, both environmental regulations and collectivist culture exert considerable influence on individuals, particularly in shaping their environmental attitudes and behaviors. Individuals within collectivist cultures tend to exhibit inclinations toward resource conservation and engagement in eco-friendly consumption practices (Dunlap and Liere, 1984; Kim and Choi, 2005). Studies by Cho et al. (2013) focusing on young populations in the United States and South Korea revealed a significant correlation between collectivist tendencies and positive environmental attitudes. Similarly, Nartova-Bochaver et al. (2022) found that respondents from collectivist cultural regions are more attuned to global climate deterioration. Moreover, research by Xue et al. (2016) conducted among participants in Beijing showcased stronger support for climate change control policies among individuals with a collectivist cultural background. Jia et al. (2017) also observed that environmental activists tend to endorse self-transcendent values, aligning closely with collectivist traits.

Beyond individual attitudes, environmental economists scrutinize how collectivism influences enterprise decision-making, particularly in environmental contexts. Studies suggest that managers rooted in collectivist cultures demonstrate a proclivity toward environmentally favorable decisions. Wang et al. (2022) discovered that companies led by CEOs from regions steeped in collectivist culture tend to make more environmentally conscious investments and R&D choices to comply with environmental regulations. Similarly, Qu (2023) identified that collectivist culture contributes to managerial decisions favoring regional green transformation. Collectivists, both at the individual level and within policymaking spheres, tend to share unified environmental protection goals and foster positive environmental attitudes. Consequently, collectivist culture, operating as an informal institutional force, can complement environmental regulatory frameworks effectively.

Secondly, Sivadas et al. (2008) classified collectivism into horizontal collectivism (HC) and vertical collectivism (VC). While both recognize the significance of interdependence, HC emphasizes individuals’ pursuit of personal goals, whereas VC individuals are more inclined to sacrifice personal objectives for the sake of the group’s goals (Triandis and Gelfand, 1998). Therefore, in regions characterized by higher levels of vertical collectivism, individuals are more willing to adhere to constraints and norms when regulations contribute to collective goals, such as emission reduction. For instance, during the COVID-19 pandemic, collectivist cultures facilitated greater adherence to government directives among local populations (Song and Choi, 2023), ensuring effective implementation of preventive measures like wearing masks (Lu et al., 2021), consequently reducing infection rates. Kumar’s (2021) global study on collectivist cultures and the severity of COVID-19 confirmed this observation. Similarly, Xue et al. (2016) found in samples from Beijing that respondents with a collectivist cultural background expressed greater support for climate change control policies compared to individualists.

Comparable evidence exists within collectivist groups, where survey research demonstrates their heightened concern for ecological conservation (Semenova, 2015; Peng et al., 2019; Naiman et al., 2023) and greater likelihood to endorse corresponding policies. Enterprises are also influenced similarly; collectivist culture impacts managerial decisions within companies (Fok et al., 2016), stimulating corporate social responsibility (Thanetsunthorn, 2015), compliance with environmental policies, and reduction of carbon emissions (Rahman et al., 2023). Therefore, we propose *Hypothesis 1a: Collectivism exhibits a negative moderating effect on the impact of environmental regulations on pollution emissions.*

### 2.2 How does collectivist culture influence pollution emissions

The impact of collectivist culture itself on pollution emissions remains inconclusive. Some cross-national studies have found a negative correlation between collectivist culture and regional environmental performance (Husted, 2005; Graafland and Noorderhaven, 2018). However, this contradicts the earlier conclusion that collectivists hold more positive environmental attitudes. We suggest several explanations for this discrepancy.

Firstly, regional environmental regulations play a significant role in identifying the relationship between collectivist culture and pollution emissions. Ho et al. (2012) found that developed Asian countries, with collectivist cultures, exhibit higher environmental awareness than North American countries but lower than European countries. This discrepancy stems from the fact that European countries tend to have more stringent regulations, whereas the United States favors self-regulation. Stringent environmental regulations better control heterogeneous regional pollution emissions.

Moreover, when controlling for similar environmental regulations, Asian countries’ collectivist culture restricts their discretionary powers, resulting in less societal oversight of corporate environmental practices compared to European countries. For example, due to its collective nature, Asian societies might be less inclined to judge or question the actions of senior executives in a company, even if these actions contradict individual values or beliefs. In a recent study, Muttakin et al. (2022) also discussed the importance of democracy in moderating the influence of culture on greenhouse gas emission intensity. Therefore, when regulatory force weakens, collectivists may engage in actions aligned with their environmental attitudes. Hence, we propose *Hypothesis 1b: Collectivism exhibits a stronger moderating effect in moderate regulations.*

Environmental regulations can be categorized into command-and-control regulations and market-based regulations (Zhao et al., 2009). Command-and-control regulations typically refer to environmental policies established and enforced by the government. On the other hand, market-based regulations involve the government designing market-based mechanisms, such as emission permits, pollution rights trading, and pollution charges, to incentivize firms to adjust their production processes and reduce pollution emissions (Hennessy and Roosen, 1999; Zhao et al., 2009). We will examine in this study whether moderate-intensity market-based policies afford greater discretion to firms in regions with collectivist cultures, enabling them to act in line with their environmental attitudes.

### 2.3 The effect of collectivist culture on pollution emissions in China

In the context of China, environmental issues exhibit a dual nature, serving as both a political achievement and cost. For local officials, resolving environmental problems can enhance their political reputation, but it also implies limitations on the operational activities of local enterprises, potentially negatively impacting regional Gross Domestic Product (GDP). This factor often significantly influences the assessment and advancement criteria for these officials. Additionally, these regulations commonly elevate production costs for enterprises, posing a challenge to the primary objective of profit maximization for shareholders.

**FIGURE 1:**
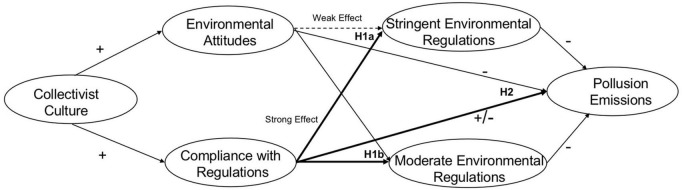
Conceptual model.

As a result, we suggest that when faced with conflicting goals between economic growth and environmental protection, economic growth tends to be prioritized as a collective interest, potentially leading to the perception that environmental protection is a secondary task that may be overlooked (Kyriacou, 2016). Furthermore, collectivism is more prone to fostering corruption issues (Mazar and Aggarwal, 2011), allowing local governments and polluting enterprises to maintain production by temporarily shutting down businesses, manipulating samples for inspection, or falsifying emission data when faced with higher-level environmental policies. Therefore, we propose *Hypothesis 2: Collectivism exhibits a positive effect on pollution emissions in China, leading to an increase in emissions.* To elucidate the interconnections between hypotheses and diverse factors, we have developed a conceptual model, depicted in Figure 1.

### 2.4 The STIRPAT model and control variables

To investigate the relationship between collectivist culture, environmental regulations, and environmental pollution, it is essential to integrate them into a unified model. Leveraging the work of Dietz and Rosa (1997), this study employed the STIRPAT (Stochastic Impacts by Regression on Population, Affluence, and Technology) model to establish a foundational regression framework. The STIRPAT model aims to explore the potential impact of factors such as population growth, economic affluence, and technological advancements on environmental effects. Over time, this model has found widespread application in studies concerning environmental pollution (York et al., 2003; Fan et al., 2006; Shahbaz et al., 2016; Vélez-Henao et al., 2019).

Ayad et al. (2023) utilized the STIRPAT model and data spanning from 1970 to 2020 to examine the influence of economic policy uncertainty on environmental quality in the Middle East and North African countries. Similarly, Sadorsky (2014) investigated the impact of urbanization on the environment using the STIRPAT model in emerging economies. The enduring use of the STIRPAT model stems from its adaptability. For instance, Wang et al. (2013) incorporated energy structure and foreign trade development into the model to analyze environmental stress in China’s Guangdong province. Adebayo et al. (2023) introduced factors like risk and renewable energy consumption into the STIRPAT model when studying the ecological footprint of MINT countries. Moreover, York et al. (2003) suggested the inclusion of an ecological resilience index in the STIRPAT model to measure the sensitivity of natural environments to various influencing factors. Ofori et al. (2023) complemented the STIRPAT model with environmental technology, environmental taxes, and institutional frameworks to validate the role of sustainable environmental policies and taxation systems in carbon emissions reduction.

In order to explore the impact of cultural variables in research, Hofstede (2001) recommends controlling for economic development. The main reason is that if economic, biological, and technological variables are more predictable then cultural indexes become redundant. Hence, this study extends the basic framework of the STIRPAT model by incorporating constructed collectivist cultural variables and regional control variables. Gross Domestic Product (GDP) per capita measures the total production of goods and services within a region, reflecting its economic strength and market size. In China, a nation with an industrial base, regions experiencing rapid GDP growth may prioritize economic development over environmental protection (He et al., 2020), influencing local individualistic culture (Hamamura, 2012), hence necessitating control. Current research widely acknowledges the nonlinear relationship between economic growth and environmental pollution (Cole et al., 1997; Stern, 2017). Thus, including the squared term of GDP in linear models is a common approach. Cai et al. (2021) proposed that China’s urbanization is linked to its ecological environment, affected by factors such as pollution control measures and economic development (Zhang et al., 2022). Furthermore, urbanization is related to regional collectivist culture (Gong et al., 2021) and hence requires inclusion in the model. Industrial structure represents the proportion of agriculture, industry, and services in the economic structure. The weight of industry and services indicates the trend of industrial structure transitioning from lower to higher forms. Mi et al. (2015) noted that with a rational industrial structure setup, energy consumption intensity can be reduced without affecting economic growth, thereby diminishing environmental pollution. Foreign direct investment (FDI) is a primary form of modern capital internationalization. For developing countries, FDI may relocate highly polluting factories to the invested country, leading to adverse environmental effects (Rafindadi et al., 2018).

### 2.5 Endogeneity of collectivist culture and environmental regulation

Existing research has demonstrated that air pollution can potentially evoke collectivist tendencies by impacting mental health (Bao et al., 2018; Li and Zhang, 2019). Hence, the connection between collectivism, environmental regulations, and pollution emissions may contain endogeneity. To address this issue, this study aims to employ instrumental variable methods. An effective instrumental variable necessitates a correlation with the original variable, and this section will focus on elucidating the theoretical background of this correlation.

Previous research has established a strong association between divorce rates and collectivism (Dion and Dion, 1996). Individualists are less inclined to sacrifice personal satisfaction for a failed marriage, even when divorce involves emotional and economic costs (Toth and Kemmelmeier, 2009). Consequently, regions with higher levels of collectivism often exhibit lower divorce rates (Hou et al., 2016; Ji et al., 2023). In this study, we will use the divorce rate per 10,000 individuals as an instrumental variable (iv1) to capture the relationship between divorce rates and collectivism.

Clan systems represent a social organization emphasizing kinship ties, shared responsibilities, and mutual assistance among family members (Peng, 2004). These systems foster social trust and facilitate collective action (Zhang and Ma, 2017). Thus, there exists a positive correlation between clan culture and the intensity of collectivist values. Consistent with prior research, we will adopt the density of genealogical records per 10,000 individuals as a proxy for clan culture (iv2).

Confucianism emphasizes the stability and harmony of family, community, and society, as well as individual responsibilities, obligations, and loyalty. These values have shaped a collective orientation within Chinese society, where individuals prioritize community interests over personal gains and believe their behavior should adhere to social norms and moral principles. Park (1998) defines Confucian collectivism as a specific dimension based on group behavior regulation, conformity, interdependence, and face-saving. To measure the strength of Confucian culture, we have chosen the number of successful candidates (also known as “Jinshi”) in the imperial examinations during the Qing Dynasty as a representative indicator. During this period, success in these exams granted individuals official positions and social status, reflecting the local population’s adherence to Confucian ideology, indirectly indicating the intensity of Confucian culture (iv3).

Lastly, Local governments typically summarize previous-year achievements, propose developmental objectives, and outline policy directions for the subsequent year in their annual reports. Hence, the frequency of environmental-related terms (iv4) in these reports signifies the government’s willingness, determination, and overall capacity in environmental governance. Moreover, as government reports precede environmental data for the year, this temporal difference mitigates concerns about timing-induced endogeneity issues (Yang and Niu, 2023).

## 3 Materials and methods

### 3.1 Baseline model

Dietz and Rosa (1997) proposed the STIRPAT model based on the IPAT framework, which categorizes the human factors influencing environmental pollution into population size, wealth, and technology. The IPAT model is typically represented as $\text{I}=\alpha \,P_{i\,t}^{b}\,A_{i\,t}^{c}\,T_{i\,t}^{d}\,e_{i\,t}$,where I, P, A, T represent environmental pollution, population, wealth, and technology, respectively, and e represents the error term. Taking the logarithm of both sides of the IPAT equation yields the STIRPAT model, which can be expressed as:


(1) $$\text{ln}\,I_{i\,t}=\alpha +b\,\text{ln}\,P_{i\,t}+c\,\text{ln}\,A_{i\,t}+d\,\text{ln}\,T_{i\,t}+e_{i\,t}$$

To examine the effect of environmental regulation on pollution reduction, we extend Model (1) and obtain the baseline model as shown in Equation (2):


(2) $$P\,I=\beta_{1}\,E\,R_{i\,t}+\beta_{2}\,L\,\_\,E\,R_{i\,t}+\beta_{3}\,c\,o\,n\,t\,r\,o\,l+u_{i}+v_{t}+\varepsilon_{i\,t}$$

Where, PI represents the environmental pollution intensity constructed using the entropy weight method. Recognizing the potential lagged effect of environmental regulation, we include both the current variable of environmental regulation (ER) and its lagged 1 year variable (L_ER) in the model. To ensure sample size and panel data balance, we selected the lagged period to be 1 year, using data from 2009, 2011, 2013, 2015, 2017, and 2019 as L_ER. Control represents a series of control variables, including economic growth, population density, technological progress, and others. The terms u and v represent the fixed effects of provinces and years, respectively.

### 3.2 Moderation effects model

The concept of moderation effect refers to the variation in the relationship between an explanatory variable and a dependent variable due to changes in a moderating variable. A basic model for moderation effect is shown in Equations (3) and (4), where D represents the explanatory variable, Y represents the dependent variable, and M is the moderating variable. With the inclusion of the moderating variable M, the marginal effect of the explanatory variable on the dependent variable changes from β_1_to $\beta_{1}^{\prime }+\beta_{3}\times M$. In the context of interaction effects, when the coefficient β_3_ > 0, it signifies that the positive influence of the explanatory variable on the dependent variable intensifies with an increase in the moderating variable. Conversely, when *β*_3_ < 0, it indicates that the positive impact of the explanatory variable on the dependent variable diminishes as the moderating variable increases. Therefore, the coefficient *β*_3_ is typically examined to assess the presence of a moderation effect within the model.


(3) $$Y=\beta_{0}+\beta_{1}\,D+\beta_{2}\,M+\varepsilon$$

(4) $$Y=\beta_{0}^{{}^{\prime }}+\beta_{1}^{{}^{\prime }}\,D+\beta_{2}^{{}^{\prime }}\,M+\beta_{3}\,M\times D+\varepsilon$$

To examine the moderating effect of collectivist culture on environmental regulations, we introduce the collectivism variable into model (2) and construct model (5) to test its moderation effect.

$$P\,I=\alpha_{1}\,C\,o\,l_{i\,t}+\beta_{1}\,E\,R_{i\,t}+\beta_{2}\,L\,\_\,E\,R_{i\,t}+\alpha_{2}\,C\,o\,l_{i\,t}\times L\,\_\,E\,R_{i\,t}$$

(5) $$+\beta_{3}\,c\,o\,n\,t\,r\,o\,l+u_{i}+v_{t}+\varepsilon_{i\,t}$$

Where Col denotes the collectivism index of each province, and *Col*×*L*_*ER* represents the interaction term between collectivism and environmental regulations.

### 3.3 Variable selection and data sources

#### 3.3.1 Dependent variables

Pollution Intensity (PI): To obtain a comprehensive assessment of pollution emissions in Chinese provinces, we curated data on three principal pollutants: industrial wastewater emissions, sulfur dioxide emissions in industrial exhaust gases, and the generation of general industrial solid waste. These data were sourced from the “China Environmental Statistics Yearbook” for the period spanning 2010–2020. Following the approach employed by Wang and Sun (2017), we applied the entropy weighting method to combine these three variables. The entropy weighting method is an objective technique that assigns weights to indicators based on the amount of information provided by each indicator’s observed values. In our study, we standardized the three pollution indicators and calculated weight coefficients based on their entropy values or redundancy degree. Ultimately, a comprehensive pollution intensity index is created by a multiple linear weighting function. This index offers a more holistic representation of pollution emissions at the provincial level compared to considering individual pollutant categories in isolation.

#### 3.3.2 Explanatory variables

Environmental regulations (ER): The quantification of environmental regulation intensity in China has been lacking a standardized measure (Tu and Chen, 2015). Previous studies have often employed alternative indicators, considering input costs and post-implementation outcomes, to evaluate environmental regulations. This study adopts environmental pollution control investment (Zhong and Chen, 2022) as a proxy for assessing the environmental regulations, utilizing data sourced from the “China Environmental Statistical Yearbook.”

Collectivism (Col): This study constructs the provincial-level collectivism variable using data from the China Family Panel Studies (CFPS)^1^. The CFPS, implemented by the Institute of Social Science Survey (ISSS) at Peking University, aims to track and collect data at the individual, household, and community levels, providing insights into the social, economic, demographic, educational, and health changes in China. The sample covers 31 provincial-level units^2^ in mainland China. The survey includes all members of the sampled households, with a sample size of approximately 16,000 households. The CFPS started in 2010 and has been conducted every year with data available up to 2020. Therefore, based on data availability, we utilize data from 2010, 2012, 2014, 2016, 2018, and 2020 to construct a panel indicator of collectivism with lag years.

Brewer and Chen (2007) decomposed collectivist culture into three dimensions: “Self-Representations,” “Beliefs,” and “Values.” Based on this classification, we selected questions from CFPS that correspond to each dimension and created a comprehensive index of collectivist culture. In the “Self-Representations” dimension, which refers to identity and emphasizes group identification, we selected the question “Are most people primarily out for themselves or willing to help others?” This question reflects respondents’ perception of the group (who are we) and measures the extent to which collectivist individuals believe that the group will provide them with assistance, indicating an interdependent self (Gorodnichenko and Roland, 2012). In the “Beliefs” dimension, which pertains to understanding social functioning, we chose the question “In today’s society, hard work brings rewards” to measure respondents’ attribution of personal achievements. Collectivist individuals are more likely to attribute achievements to external factors (Oyserman et al., 2002). In the “Values” dimension, we selected the question “Trust in local officials/government” as an indicator. Collectivist individuals tend to prioritize collective interests over personal ones and believe that collective decision-making can achieve greater common benefits. Since CFPS does not provide specific city information for each sample household to protect respondents’ privacy, we constructed a provincial-level collectivism intensity variable based on the province of the sample. For each year’s data, we standardized and combined the three dimensions of collectivism using max-min normalization to obtain a single collectivism score for each sample. Finally, we averaged the scores based on the province to obtain annual provincial-level collectivism indicators.

**FIGURE 2:**
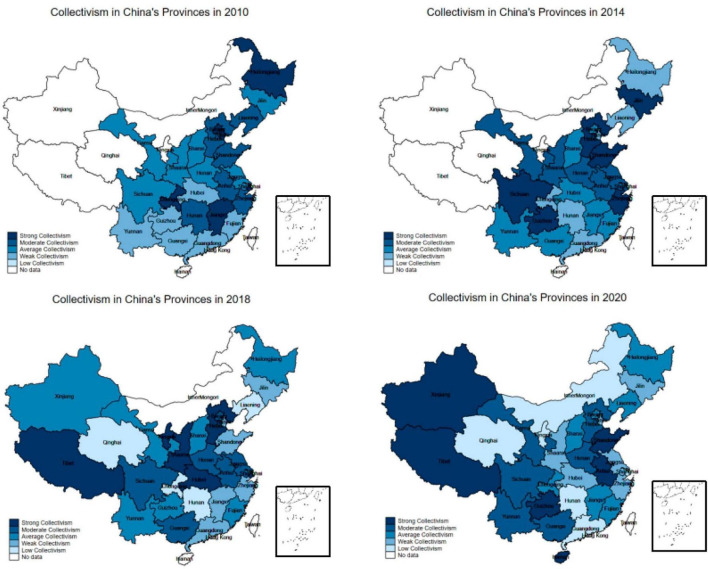
The distribution of collectivism intensity in China from 2010 to 2020. The map template is from the Map Technology Review Center of the Ministry of Natural Resources (GS (2022) 4312).

We selected collectivism indicators from the years 2010, 2014, 2018, and 2020 and created four maps of China to illustrate the spatial-temporal heterogeneity of collectivism changes from 2010 to 2020. The results are shown in Figure 2. The regional analysis reveals a distinct north-south divide in collectivist culture. Provinces in the northern region, such as Beijing, Hebei, Shandong, Shanxi, Henan, and Anhui, exhibit a stronger prevalence of collectivism compared to provinces in the southern region. This can be attributed to the influence of traditional Confucian culture, which emphasizes collective interests and social responsibility, and originated in the northern region of China. The agricultural nature of the northern region, with its emphasis on collective cooperation and rural community support, further reinforces the prevalence of collectivism in these areas. In terms of temporal changes, the southeastern coastal regions, including Shanghai, Zhejiang, and Jiangsu, demonstrate a gradual decline in collectivism. These regions, characterized by rapid modernization and urbanization, have experienced increased social mobility and reduced interpersonal connections. As a result, individuals in these areas tend to prioritize personal economic interests and competition, leading to a weakening of the collectivist culture. Moreover, the year 2020 shows a notable increase in overall collectivism across China compared to 2018. This finding aligns with the pathogen-prevalence hypothesis, which suggests that the COVID-19 pandemic has prompted individuals to embrace collectivist values as a means of protecting themselves and others from perceived threats.

#### 3.3.3 Control variables

Based on previous studies, we included several control variables in our analysis. Per capita GDP (pgdp) and its squared term (sqr_pgdp) were considered as proxies for economic development. The urbanization rate (Urb), calculated as the proportion of urban population to total population within each province, was included to account for the level of urbanization. The composition of GDP between the secondary and tertiary sectors was captured by the ratio of the second industry to the third industry (IS). Moreover, we constructed an indicator of industrial upgrading (ISU) by comparing the increment of GDP in the tertiary industry to that in the second industry. Consistent with the “pollution haven” hypothesis (Tang et al., 2022), we used the annual realized amount of foreign investment as a measure of foreign direct investment (FDI). The data sources for these variables were the “China Statistical Yearbook” and the National Bureau of Statistics of China. For a more detailed description of the relevant variables, please refer to Table 1.

**TABLE 1:** Variable definitions and data sources.

Types of variables	Abbreviation	Variable	Data sources
Key variables	PI	Environmental pollution emission intensity	«China Environmental Statistical Yearbook»
	ER	Environmental regulation	
	Col	collectivism	China Family Panel Studies(CFPS)
Control variables	pgdp	Per Capita GDP	«China Statistical Yearbook» and China National Bureau of Statistics
	sqr_pgdp	Square of per capita GDP	
	Urb	Urbanization rate	
	IS	Industrial structure	
	ISU	Industrial structure upgrading	
	FDI	Foreign direct investment	
Instrumental variables	iv1	Divorce rate	China National Bureau of Statistics
	iv2	Clan genealogical density	«General Catalogue of Chinese Genealogy»
	iv3	Number of Jinshi in the Qing Dynasty	«Index of Ming and Qing Jinshi Inscription Steles»
	iv4	The frequency of vocabulary related to environmental protection appearing in local government work reports	The Government Work Report of provinces
Mediating variables	inno	Proportion of green patent applications to total patent applications	«China Statistical Yearbook»
Other variables	Col2	The number of Monuments for Virtuous Women in each province of China	«History of Ming Dynasty⋅Biographies of Chaste Female»«The draft of Qing History»
	Col3	Number of Existing Confucian Temples in China	China Research Data Service Platform(CNRDS)
	PI2	Environmental pollution emission intensity	«China Statistical Yearbook on Environment»
	NO_X_	Nitrogen oxide	«China Energy Statistical Yearbook»

Due to the limitations of the CFPS questionnaire, we used data from 2010, 2012, 2014, 2016, 2018, and 2020 to construct the collectivism index with gap years. For provinces including Xinjiang, Tibet, Inner Mongolia, Ningxia, Qinghai, and Hainan, data with missing values before 2018 were excluded from the regression analysis. To address heteroscedasticity, we logarithmically transformed variables such as environmental pollution intensity, environmental regulations, GDP, and foreign direct investment. Please refer to Table 2 for more details. To improve model stability and interpretability while avoiding multicollinearity, all interaction terms in the model were centered.

**TABLE 2:** Descriptive statistics of variables.

Variable	Mean	SD	Min	Max	N	Unit
PI	0.297	0.183	0.000	0.728	186	–
ER	11.654	1.326	6.165	14.164	186	ln Ten thousand yuan
Col	2.001	0.221	1.000	2.667	163	–
pgdp	10.720	0.484	9.464	12.009	186	Ten thousand yuan/person
sqr_pgdp	115.148	10.438	89.559	144.206	186	Ten thousand yuan^2^/person^2^
Urb	0.574	0.135	0.227	0.893	186	Urban population/total population
IS	0.996	0.351	0.189	2.002	186	Output value of Secondary sector of the economy/output value of Tertiary sector of the economy
ISU	0.892	0.295	0.191	1.757	186	Newly increased output value of Tertiary sector of the economy/newly increased output value of Secondary sector of the economy
FDI	11.101	1.585	6.280	14.825	186	Ln Ten thousand dollars

## 4 Results of empirical analysis

### 4.1 Baseline regression analysis

Table 3 (1) reports the regression results of the baseline model examining the emission reduction effect of environmental regulations. The findings demonstrate a significant long-term inhibitory effect of environmental regulations on pollution emissions, albeit with a lag effect. Specifically, both the current environmental regulations (ER) and lagged environmental regulations (L_ER) coefficients are significant at the 1% level with opposite signs. An increase of 1% in current environmental regulations leads to a 0.048% increase in pollution emissions for the same year, whereas a 1% increase in lagged environmental regulations results in a 0.085% decrease in pollution emissions. This outcome suggests a combined effect of the “green paradox” and “closure emissions reduction.” The “green paradox” effect arises as government-regulated environmental regulations force polluting enterprises to adopt emission reduction measures. Small-scale enterprises facing financial constraints may struggle to comply with the emission standards, potentially resulting in production reductions or closures. Consequently, these enterprises may strategically increase pollution emissions during the window period between policy announcement and implementation to minimize losses. On the other hand, large-scale enterprises with sufficient resources tend to transition to cleaner production methods and invest in clean energy and green technologies to achieve emission reduction goals. However, such adjustments may require more time for implementation. Furthermore, there might be a reverse causality between current environmental regulations and pollution emissions. Regions experiencing severe environmental pollution may prompt government authorities to improve the environment through policy interventions to showcase their performance. Consequently, the combined effect of these factors can lead to short-term increases in pollution emissions, while promoting long-term emission reductions.

**TABLE 3:** Results of the baseline regression and moderation regression.

	(1)	(2)	(3)
	**Model1**	**Model2**	**Model3**
**VARIABLES**	**PI**	**PI**	**PI**
Col		0.4062***	0.6073***
		(3.90)	(5.21)
lnER	0.0479*	0.0270	0.0231
	(1.80)	(1.02)	(1.20)
L_lnER	−0.0845*	−0.0767***	−0.0674***
	(−1.95)	(−2.45)	(−2.20)
ColxL_lnER			−0.0064***
			(−3.84)
lnpgdp	2.0470	3.2580***	5.0535***
	(1.15)	(1.82)	(2.92)
sqr_lnpgdp	−0.1147	−0.1656***	−0.2451***
	(−1.44)	(−2.11)	(−3.12)
IS	0.0522	0.4036**	0.5356***
	(0.25)	(2.70)	(2.89)
ISU	0.1479	−0.3141**	−0.4015**
	(0.50)	(−2.13)	(−2.53)
Urb	3.7180**	1.4705	1.5408
	(2.68)	(0.99)	(1.03)
FDI	0.0083	−0.0359	−0.0513
	(0.23)	(−0.74)	(−1.20)
N	163	163	163
R^2^	0.206	0.402	0.589
Provinces fixed effect	Y	Y	Y
Year fixed effect		Y	Y

***, **, and * indicate significant at significance levels of 1, 5, and 10%, respectively.

### 4.2 The moderation effects of collectivism

According to equation (5), we constructed a moderated effect model that includes the collectivism variable. The regression results in column (3) of Table 3 show that, after introducing the collectivism variable, the coefficient of environmental regulations (L_ER) remains significant at the 5% level, but slightly reduced compared to the previous model. Considering that we centered the interaction terms, indicates that when collectivism is at its average level, i.e., when ColxL_ER is 0, a 1% increase in environmental regulation intensity will lead to a reduction in pollution emissions by approximately 0.07%.

The results confirm Hypothesis 1a, indicating that collectivism plays a negative moderating effect in the relationship between environmental regulations and pollution emissions. The interaction term ColxL_ER is significant at the 1% level and negative, indicating that as the level of collectivism increases, the emission reduction effect of environmental regulations also increases. In addition, the variable of collectivism (Col1) is positively correlated with pollution emissions at the significance level of 1%, confirming hypothesis 2 that regional collectivism has a positive effect on pollution emissions. The regression results for the control variables reveal that the relationship between environmental pollution and economic growth, as indicated by the coefficients of GDP growth (pgdp) and its quadratic term (sqr_pgdp), still follows an inverted “U” shape and has not yet reached the turning point. This finding confirms that China’s economic growth over the past decade has come at the expense of the environment. Additionally, the coefficients for industrial structure (IS) and industrial structure upgrading (ISU) validate the fact that the service sector is more environmentally friendly compared to the manufacturing sector. This further supports the positive impact of upgrading the industrial structure on improving environmental quality.

### 4.3 Robustness analysis

To ensure the robustness of our regression results, we conducted rigorous robustness tests. We employed principal component analysis to construct a new dependent variable using nitrogen oxide emission intensity as an alternative pollution indicator. Furthermore, we considered additional measures of collectivism to broaden the sample size and validate the moderating effect. These measures strengthen the reliability and robustness of our research findings.

#### 4.3.1 Replace the explained variable

Principal Component Analysis (PCA) is a method commonly used to transform high-dimensional data into a lower-dimensional representation through linear transformations. Similar to the entropy weighting method, PCA aims to create a comprehensive pollution emission index by preserving the essential components while reducing the dimensionality of the variables. Instead of using entropy values as weights, PCA determines the weights based on the eigenvalues of the covariance matrix between variables. In our study, we applied PCA to reduce the dimensionality of the variables, namely industrial wastewater emissions, sulfur dioxide emissions in waste gases, and general industrial solid waste generation (Chao and Ren, 2011). This allowed us to generate a new composite index called PI2, which was then included as the dependent variable in the regression model. The regression results presented in Table 4 (2) indicate that the signs and coefficients of the core explanatory variables and moderating variables remain stable and statistically significant at a significance level of 5%.

**TABLE 4:** Results of robustness analysis.

	(1)	(2)	(3)	(4)	(5)
	**Model3**	**Replace the dependent variable**	**Replace the dependent variable**	**Replace the moderator**	**Replace the moderator**
**VARIABLES**	**PI**	**PI2**	**NOx**	**Col2**	**Col3**
L_ER	−0.0674** (−2.20)	−0.0796*** (−2.80)	−0.0310 (−1.21)	−0.0106* (−1.78)	−0.0090* (−1.67)
Col	0.6073*** (5.21)	0.2465*** (2.98)	0.1398** (2.40)		
ColxL_ER	−0.0064*** (−3.84)	−0.0054** (−2.56)	−0.0131** (−2.29)		
Col2				0.0001** (2.55)	
Col2xL_lnER				−0.0002*** (−2.64)	
Col3					0.0001*** (4.66)
Col3xL_lnER					−0.0001** (−2.30)
Control variables	Y	Y	Y	Y	Y
Provinces fixed effect	Y	Y	Y	Y	Y
Year fixed effect	Y	Y	Y	Y	Y
N	163	163	163	310	308
R^2^	0.589	0.511	0.871	0.355	0.304

***, **, and * indicate significant at significance levels of 1, 5, and 10%, respectively.

Nitrogen oxides (*NO*_*x*_) are air pollutants composed of nitrogen and oxygen elements, including nitric oxide (*NO*) and nitrogen dioxide (*NO*_2_). They are mainly generated from human activities such as combustion processes, industrial production, and transportation (Heck, 1999). Therefore, nitrogen oxides serve as important surrogate indicators for pollution emissions, particularly industrial pollution emissions. We compiled carbon oxide emission indicators from the “China Energy Statistical Yearbook” and included them in the model. The regression results in Table 4 (3) demonstrate that the signs and coefficients of the moderating variables remain stable and statistically significant at a 5% significance level.

#### 4.3.2 Replace the moderating variable

The Monument for Virtuous Women is a commemorative architectural structure in Chinese traditional culture (Zhao et al., 2016), which honors and memorializes women known for their moral integrity and adherence to virtuous principles (Wu, 2015). During the late 18th to the 19th centuries, Chinese societal expectations placed significant emphasis on individuals upholding filial piety and moral principles. Women who demonstrated exemplary behavior in maintaining moral standards, particularly in their roles as wives and daughters-in-law, were celebrated and memorialized through the construction of these monuments. The presence of the Monument for Virtuous Women in a region signifies the cultural importance placed on moral values and virtuous behavior. It reflects the collective recognition and reverence for individuals who uphold traditional ethical principles and contribute to the preservation of social harmony and moral integrity. The data on these monuments used in this study were obtained from historical records and texts, including “The Biographies of Exemplary Women” within “History of Ming Dynasty- Biographies of Chaste Female” and “The draft of Qing dynasty history.”

The Confucius Temple, a significant site for venerating Confucius and propagating Confucian philosophy, stands as a principal channel for disseminating Confucian culture. Central to Confucian ideology is the emphasis on individuals pursuing social harmony, prioritizing collective interests, and fulfilling societal roles and responsibilities. In nations and regions deeply influenced by Confucianism, there is a tendency to prioritize collective welfare (Morling and Lamoreaux, 2008; Ma X. R. et al., 2016), respect elders and traditional authorities, and value social order and public morality. Since the Han Dynasty, the Confucius Temple has served the societal function of “civilizing the populace” (Yeo, 2014). Over ensuing centuries, it has continued to propagate Confucian ideology, transcending ethnic boundaries (Han, 2007) to become a primary avenue for disseminating Confucian culture. The symbiotic relationship between the Confucius Temple and Confucian culture, coupled with the robustness of historical data, has led numerous scholars to consider the quantity of Confucius Temples as an indicator of the strength of local Confucian culture and collectivist values (Du, 2015, 2016; Jin et al., 2017). The data on Confucius Temples utilized in this study were sourced from the China National Research Data Center.

Due to the limited availability of CFPS data, the previously constructed collectivism index (Col) in this study only covers a span of 6 years. To further examine the robustness of the collectivism moderation effect, we expanded our analysis by incorporating data on monuments for virtuous women and existing Confucius Temples, and interacted them with yearly dummy variables from 2010 to 2020. This allowed us to construct two additional collectivism indicators, Col2 and Col3, spanning a period of 11 years from 2010 to 2020.

Analyzing the regression results in Tables 4 (4) and (5), we find that even after these variable adjustments, the interaction term between collectivism and environmental regulations remains significantly negative at the 1% level. Moreover, the effect of collectivism on pollution emissions shows significant positive associations at the 5 and 1% levels, respectively. These findings affirm the robustness of the moderation effect and provide further support for Hypotheses 1a and 2.

### 4.4 Endogeneity analysis

To ensure the accuracy of the impact of collectivism and to address potential endogeneity concerns, this study utilized appropriate instrumental variables in the analysis. While the fixed effects model employed in the initial regression helps mitigate endogeneity to a certain extent, there’s a possibility of a reverse causality existing between collectivism and pollution emissions. For instance, a worsening ecological situation might elevate public attention toward environmental concerns, leading to the adoption of collectivist values (Fincher et al., 2008; Bao et al., 2018). To tackle this challenge, this study introduced divorce rates, provincial genealogy records, and the number of successful candidates in the Qing Dynasty’s imperial examinations as instrumental variables. Here, we used the divorce rate per 10,000 individuals as an instrumental variable (iv1), and similarly, we employed the density of genealogical records per 10,000 individuals as a proxy for clan culture (iv2) and the count of “Jinshi” (iv3) as an indicator of Confucian culture.

Furthermore, potential reverse causality issues might exist concerning environmental regulations (Wang et al., 2019). Hence, this study utilized the frequency of environment-related terms in local government work reports as an instrumental variable for environmental regulations. These instrumental variables enhance the identification strategy and bolster the robustness of the findings. Building upon the methodologies of Chen and Chen (2018) and Yin and Wu (2021), we adopted the frequency of environment-related terms in local government work reports as an instrumental variable (iv4) for environmental regulations.

Given the potential endogeneity in both collectivism and environmental regulations, we separately paired the three collectivism variables (iv1, iv2, iv3) with the environmental regulation variable (iv4) and employed them as instrumental variables in a two-stage least squares (2SLS) regression. The Kleibergen-Paap rank (rk) Wald test statistics for the regression models all exceeded 10, rejecting the null hypothesis of weak instrumental variables. The outcomes in Table 5 (2) to (4) demonstrate that, after addressing endogeneity concerns, the moderating effects of collectivism are amplified, and the regression outcomes remain robust compared to the baseline model.

**TABLE 5:** Results of instrumental variable regression.

	(1)	(2)	(3)	(4)
	**Model 3**	**iv1&iv4**	**iv2&iv4**	**iv3&iv4**
**VARIABLES**	**PI**	**PI**	**PI**	**PI**
L_ER	−0.0674** (−2.20)	0.0669 (0.41)	−0.0808 (−0.41)	−0.7892** (−2.03)
Col	0.6073*** (5.21)	0.9561** (1.98)	1.0716** (2.24)	1.6257 (1.44)
ColxL_ER	−0.0064*** (−3.84)	−0.0096** (−2.08)	−0.0112** (−2.16)	−0.0185* (−1.82)
Control variables	Y	Y	Y	Y
Provinces fixed effect	Y	Y	Y	Y
Year fixed effect	Y	Y	Y	Y
Kleibergen-Paap rank (rk) Wald statistic		11.12	8.68	13.26
N	163	163	163	163

***, **, and * indicate significant at significance levels of 1, 5, and 10%, respectively.

### 4.5 Heterogeneity analysis of environmental regulations

In light of the growing importance of market-based environmental regulations within China’s regulatory framework (Xu and Sun, 2023), this section endeavors to validate *Hypothesis 1b* by investigating how collectivism moderates the effects within this specific environmental regulatory structure.

We measure market-based environmental regulations using the annual pollution fees levied in each province, obtained from the “China Environmental Yearbook”^3^ and the “China Tax Yearbook.” (see text footnote 3) Although China transitioned from pollution fees to pollution taxes in 2018 for pollution-intensive industries, the implementation followed a “smooth transition” principle. The entities that previously paid pollution fees became taxpayers for the environmental protection tax, and the tax rates were designed to avoid imposing additional financial burdens on businesses. Therefore, we believe that this reform would not significantly affect the data for 2020, considering the use of fixed-effect models.

The regression results in Table 6 demonstrate that market-based environmental regulations (−0.101) have a more pronounced effect in reducing pollution emissions compared to policy-based environmental regulations (−0.067). Market-based regulations utilize flexible incentive mechanisms that can optimize resource allocation and foster the autonomy of enterprises. These findings align with previous research findings (Feng Y. et al., 2023). In terms of the moderation effect, collectivism exhibits a stronger influence on market-based environmental regulations. The effectiveness of market-based regulations relies on cooperation among market participants, and collectivism can effectively facilitate collaboration and coordination among stakeholders, thereby enhancing policy implementation and effectiveness.

**TABLE 6:** Heterogeneity analysis results for market-based environmental regulations.

	(1)	(2)
	**Model3**	**heterogeneity**
**VARIABLES**	**ER**	**ER2**
Col	0.6073*** (5.21)	0.4015*** (4.46)
L_lnER	−0.0674** (−2.20)	
ColxL_lnER	−0.0064*** (−3.84)	
L_lnER2		−0.1006*** (−3.42)
ColxL_lnER2		−0.0053*** (−2.86)
lnpgdp	5.0535*** (2.92)	3.8031*** (3.17)
sqr_lnpgdp	−0.2451*** (−3.12)	−0.1922*** (−3.57)
IS	0.5356*** (2.89)	0.5201*** (4.09)
ISU	−0.4015** (−2.53)	−0.3553** (−2.45)
Urb	1.5408 (1.03)	1.9324** (2.04)
Provinces fixed effect	Y	Y
Year fixed effect	Y	Y
N	163	163
R^2^	0.589	0.402

*** and ** indicate significant at significance levels of 1% and 5% respectively.

### 4.6 Mechanism analysis of the impact of collectivism on environmental regulations

Existing research consistently suggests a strong link between culture and innovation (Fiordelisi et al., 2019; Wang et al., 2021). Thus, in this section, we aim to examine whether green innovation serves as a mediating mechanism for the moderating effect of collectivism on environmental regulations.

We employs the proportion of green patent applications to total patent applications as an indicator of green innovation (Inno). Previous research has found that patent indicators can control for unobservable factors in macroeconomics (Popp, 2006), and patent technologies are likely to have an impact on firms during the application process. Therefore, green patent applications effectively reflect a firm’s current level of green innovation.

Drawing on the method proposed by Baron and Kenny (1986), we used a stepwise approach to examine whether green innovation serves as a mediating channel for the moderating effect of collectivism. Firstly, we regressed green innovation as the dependent variable. The results in Table 7 (1) show a significant positive correlation between collectivism and green invention. It is worth noting that there is a slight negative relationship between environmental regulations and green innovation, suggesting the existence of a “crowding-out effect” (Link, 1982; Roediger-Schluga, 2003) in the measured environmental regulations. When firms face stricter environmental regulations, they tend to reallocate resources, such as purchasing more environmentally friendly equipment or adopting stricter emission control measures. These additional costs may reduce the firm’s investment in research and development, resulting in a certain degree of negative impact on green innovation.

**TABLE 7:** Results of mechanism analysis for green innovation.

	(1)	(2)	(3)
	**Step 1**	**Step 2**	**Step 2 (iv)**
**VARIABLES**	**Inno**	**PI**	**PI**
Inno		−0.0561* (−1.97)	−4.2929* (−1.73)
Col	0.0581*** (5.57)	0.2623** (2.65)	1.7819** (2.36)
L_lnER	−0.0081** (−2.66)	−0.0410 (−1.48)	0.1555 (0.49)
ColxL_lnER	−0.0007*** (−2.84)	−0.0034* (−1.72)	−0.0171** (−2.29)
Observations	150	150	150
Provinces fixed effect	Y	Y	Y
Year fixed effect	Y	Y	Y
R^2^	0.507	0.344	
N	25	25	25

***, **, and * indicate significant at significance levels of 1, 5, and 10%, respectively.

To examine the effect of green innovation (Inno) on pollution emissions, we included it as an explanatory variable in the baseline model. The regression results in Table 7 (2) demonstrate that green innovation effectively reduces pollution emissions, and the absolute value of the moderating effect of collectivism decreases compared to the original model, indicating that green innovation serves as an effective mediating channel for the moderation effect. To address potential endogeneity concerns and accurately identify the emission-reducing effect of green innovation, we re-estimated the model using instrumental variables for collectivism and environmental regulations (iv3 and iv4). The results in Table 7 (3) further validate the pollution-reducing effect of green innovation.

## 5 Discussion

With the growing environmental awareness at both societal and individual levels, formal and informal institutional factors related to environmental regulations continue to garner increasing attention. Current studies on informal institutional aspects of environmental regulations predominantly focus on elements like religion, clans, trust, local identity, and individual consciousness, with limited attention to the impact of collectivist cultural orientation on environmental regulations. From a cultural perspective, this research investigates the moderating effects of collectivist culture on different types of environmental regulations and their underlying mechanisms.

The findings of this study indicate, firstly, that environmental regulatory policies exhibit a significant positive impact on environmental pollution during their implementation period, yet with noticeable lagged effects on emission reduction. This implies that the effects of environmental regulatory measures become evident only after a certain period post-implementation. This conclusion aligns with the research findings of Lu et al. (2022), who discovered complex effects of environmental regulatory policies on carbon emissions across different stages, displaying a reverse U-shaped relationship at specific emission levels. Specifically, this manifests as the “green paradox” effect at certain stages, followed by a transition to the “emission reduction” effect. The reason behind this phenomenon might be attributed to the government’s regulatory approach, which often involves setting emission limits that compel polluting enterprises to adopt measures to reduce emissions. Enterprises failing to meet these standards might face closure, which could lead smaller polluting enterprises with limited funds to pay emission fees or implement pollution control techniques to reduce production or even shut down. Under this pressure, smaller entities may increase pollution emissions during the window period between the announcement and enforcement of environmental policies to mitigate losses, resulting in short-term emission escalation. On the other hand, larger, more financially robust enterprises tend to change their production methods by adopting clean energy and green equipment to reduce pollution emissions. Although these measures effectively reduce emissions in the long term, they might not promptly alter the existing production models in the short term. Furthermore, there might be a certain reverse causality between current environmental regulations and pollution emissions. Regions experiencing severe environmental pollution might incentivize governmental departments to improve the environment through policy instruments to gain performance accolades. Consequently, environmental regulations might lead to short-term increases in pollution emissions due to various factors; however, in the medium to long term, these regulations could drive a reduction in pollution emissions.

Secondly, the research findings demonstrate a positive relationship between the intensity of collectivist culture and pollution emissions, confirming Hypothesis 2, aligning with evidence from cross-national studies. Ioannou and Serafeim (2012) propose that granting autonomy to economic entities is a critical factor for them to demonstrate environmentally conscious behaviors in alignment with local cultural values. In individualistic cultures, this environmental behavior is reflected in corporations undertaking explicit and voluntary environmental initiatives, whereas in collectivist cultures, this behavior is represented by corporations aligning with externally imposed environmental policies. However, when regulatory authority becomes excessively stringent, or completely suppresses the discretionary power of economic entities, the impact of culture on corporate behavior may diminish or distort. Research by Yu and Gao (2015) in China suggests that strict environmental regulations by governmental bodies over official economic activities might prompt economic sectors to shift certain economic activities into the hidden economy (Frey and Weck-Hanneman, 1984).

China is characterized by a dominant culture of vertical collectivism and a high degree of power distance (Hofstede and Minkov, 2014). Individuals perceive themselves as part of a collective and consider inequality among members as a natural element of social order (Ma J. et al., 2016). Cho et al. (2013) discovered a negative impact of vertical collectivism on environmental attitudes, possibly due to a partial alignment of personal environmental attitudes with collective interests. Vertical collectivist culture encourages prioritizing collective interests and acknowledges status disparities within the group. When the collective interest involves sustaining production (thus maintaining pollution emissions) rather than reducing emissions, decisions might be made to shift production to informal economic activities. In such scenarios, the collectivist variable encompasses measurements of the informal economy, hence correlating positively with pollution emissions.

Thirdly, when the orientation of collectivist culture interacts with environmental regulation, the symbol turns negative, validating Hypothesis 1a. The collectivist cultural orientation plays a negative moderating effect on the impact of environmental regulations on pollution emissions, indicating that an increase in collectivism at the mean level will significantly enhance the emission reduction effect of environmental regulations. This conclusion aligns with some existing research theories. Collectivism, as a cultural background, effectively influences individuals and groups in a region, including managers in pollution emission sectors and employees engaged in actual production activities, by strengthening their willingness to comply with environmental regulatory policies (Rahman et al., 2023), and by enhancing execution strength, thereby reducing pollution emissions. Furthermore, previous studies (Xue et al., 2016; Kaplan Mintz and Kurman, 2020; Qu, 2023) have also demonstrated that collectivist groups have a higher ecological consciousness, incline toward green investments, research and recycling behaviors. When faced with mandatory government environmental regulations, highly collectivist groups tend to strictly adhere to these policies and abandon personal interests, making environmental policies more effective.

However, some studies reveal contradictory findings in this dimension (Ioannou and Serafeim, 2012; Eom et al., 2016; Graafland and Noorderhaven, 2018). These studies find a significant positive association between individualism and environmental performance. Despite seeming contradictory, Cho et al. (2013) indicated that both horizontal individualism and horizontal collectivism have a positive relationship with environmental attitudes. In horizontal individualism culture, individuals believe they benefit from environmentally friendly behavior and consider the formation of a societal consensus on environmental protection crucial. And in horizontal collectivist cultures, individuals typically see themselves as part of a group, and their behavior, decisions, and interests are often influenced by the expectations of the entire group or society. Thus, individuals in both cultures may comply with environmental protection regulations based on personal or collective interests. This study primarily emphasizes that under strict environmental protection policies, individuals within collectivist cultures may strengthen environmental regulations’ energy-saving and emission reduction effects by strictly adhering to the rules.

Moreover, compared to command-and-control regulations, collectivism has a more significant moderating effect on moderate market-based environmental regulations, confirming Hypothesis 1b. The moderating effect of collectivism varies under different types of environmental regulations, where overly stringent regulations limit the discretion of enterprises, thereby reducing the impact of collectivist culture. This conclusion aligns with previous research findings (Feng Q. et al., 2023), indicating that the effectiveness of incentive-based environmental regulations relies on cooperation among market individuals, and collectivist culture can effectively promote cooperation and coordination among various stakeholders, thereby enhancing the strength and effectiveness of policy implementation.

Lastly, the analysis of mechanisms validates that within the context of regional collectivist culture, green innovation emerges as a pivotal mechanism influencing pollution emissions. This phenomenon stems from the substantial impact of collectivist culture on both corporate leadership and the workforce, thereby fortifying green innovation through dual perspectives: strategic decision-making (Wang and Gao, 2022) and practical execution (Kong et al., 2017). The fabric of collectivism augments collaboration and trust among employees (Brockman et al., 2018), diminishing the potential for knowledge leakage and opportunistic behaviors, such as free-riding, within internal collaborations. This reduction in internal innovation hurdles notably enhances the efficacy of implementing green innovations within corporate environments. Furthermore, the conspicuous positive correlation observed between collectivism and green innovations reaffirms the notion that collectivist groups exhibit heightened ecological consciousness and a proclivity toward investments in green initiatives, including research and development. This conclusion further underscores the intricate pathways through which culture intertwines with endeavors toward environmental preservation.

## 6 Conclusion

Given the escalating environmental challenges, China is tasked with a significant mission of pursuing a green transformation. The Chinese government has implemented a series of environmental policies aimed at strengthening environmental protection and optimizing ecological sustainability in response to these pressing challenges. While prior research has primarily focused on examining environmental regulations through the government actions, the regulatory role of informal institutions remains an area that requires further exploration. This study aims to contribute to the existing literature by delving into the impact of Chinese collectivist culture on environmental regulations.

To capture the temporal and spatial heterogeneity of Chinese collectivism, this study constructs a measure of collectivist tendencies for 31 provinces in China from 2010 to 2020, utilizing data from the esteemed “China Family Panel Studies (CFPS).” Based on this measure, a panel model incorporating pollution emissions intensity and environmental regulation strength is developed. Employing a panel fixed-effect model, this study examines the moderating effects of collectivist cultural intensity on different types of environmental regulations, shedding light on the underlying mechanisms at play. Various robustness tests, including sensitivity analyses and instrumental variable approaches, are employed to enhance the robustness and validity of the regression results.

However, it is important to acknowledge the limitations of our study. Firstly, the availability of CFPS data restricted us to constructing an unbalanced panel dataset spanning only 6 years, which may have implications for the robustness of our findings. While we have undertaken robustness tests by expanding the sample through the replacement of explanatory variables, further validation of our results is warranted. Secondly, the construction of provincial-level collectivism indicators relied on using provincial-level proxies, as specific city/district-level information for the samples was not accessible. Consequently, this approach may not fully capture the regional heterogeneity of collectivism within provinces, potentially overlooking valuable information on collectivism’s variation at the city level. Lastly, the measurement of hidden economic activities is inherently challenging, requiring extensive efforts to accurately quantify. In this study, we did not investigate the impact of collectivism on pollution emissions through the channel of the hidden economy, as measuring the impact of collectivism on pollution emissions through the hidden economy would require significant additional work. And this will be one of our future research directions.

In conclusion, this study has provided insights into the impact of collectivist culture on environmental regulations from the perspective of informal institutions, thereby holds various theoretical significances. Firstly, it optimized the construction methodology of collectivist indicators, establishing a continuous provincial-level collectivist index for China, thereby more accurately depicting the spatiotemporal variations in Chinese collectivism. Secondly, the research focused on exploring the regional collectivist culture’s regulatory role in formal environmental regulations from the perspective of informal institutions, substantiating conclusions through multifaceted validations. Thirdly, by delving into collectivist culture, this study elaborated extensively on the mechanisms of its influence on diverse types of environmental regulations, providing a profound analysis of how informal institutions shape formal environmental regulations in practical settings. Finally, this research expanded the existing findings on the impact of collectivist culture on regional pollution emissions by incorporating pertinent empirical evidence from China.

The conclusions drawn from this study also offer potential insights for policy formulation and implementation. Firstly, the outcomes of this study could serve as practical guidance for governments in tailoring relevant environmental policies according to local contexts. Given the cultural diversity in China, governments could finely tune environmental policies to maximize the positive impact of cultural traits on environmental regulations, thereby enhancing policy efficacy. Secondly, this study highlights the impact of collectivist culture on pollution emissions through fostering green innovation, offering practical recommendations for policies encouraging green technology innovation. Governments and businesses could incentivize team collaboration and innovative spirit to drive advancements in environmental technology, thereby facilitating sustainable environmental development. Lastly, recognizing the potential time-lag effects in environmental regulation policies, governments should pay closer attention to implementation delays when formulating such policies, aiding continual improvements and optimizations to enhance the actual effectiveness of environmental policies.

## Data availability statement

The original contributions presented in this study are included in this article/supplementary material, further inquiries can be directed to the corresponding author.

## Author contributions

LZ: Conceptualization, Data curation, Formal analysis, Methodology, Software, Writing – original draft, Writing – review and editing. MZ: Methodology, Visualization, Writing – review and editing. JJ: Data curation, Formal analysis, Visualization, Writing – original draft. XP: Formal analysis, Software, Writing – original draft. JZ: Writing – review and editing. SY: Conceptualization, Supervision, Writing – review and editing.

## Footnotes

1
http://www.isss.pku.edu.cn/cfps/en/index.htm

2 Due to practical constraints in conducting the survey, data for the regions of Xinjiang, Tibet, Inner Mongolia, Ningxia, Qinghai, and Hainan before 2018 have partial missing values.

3
http://cnki.nbsti.net/CSYDMirror/Trade/yearbook/single/N2020100004?z=Z024
